# Neuromotor Development Evaluation of Preterm Babies Less than 34 Weeks of Gestation with Bayley III at 18-24 Months

**DOI:** 10.1155/2020/5480450

**Published:** 2020-10-20

**Authors:** Lida Bulbul, Gizem Kara Elitok, Ebru Ayyıldız, Dilek Kabakcı, Sinan Uslu, Gülsen Köse, Semra Tiryaki Demir, Ali Bulbul

**Affiliations:** ^1^University of Health Science, Department of Pediatrics, Bakırköy Sadi Konuk Education and Research Hospital, Istanbul, Turkey; ^2^University of Health Science, Sisli Hamidiye Etfal Education and Research Hospital, Department of Pediatrics, Istanbul, Turkey; ^3^University of Health Science, Sisli Hamidiye Etfal Education and Research Hospital, Department of Pediatric Ophthalmology, Istanbul, Turkey

## Abstract

**Objectives:**

To assess and evaluate the risk factors affecting the neuromotor development of preterm babies at corrected age 18 to 24 months.

**Methods:**

Preterm babies ≤ 34 weeks of gestational age (GA) who were born in our hospital between 2011 and 2014 were prospectively included in the study. Prenatal, perinatal, and postnatal features of the babies were recorded. Bayley Scales of Infants and Toddler Development, Third Edition (Bayley-III), was applied at corrected age 18 to 24 months.

**Results:**

All data of 96 babies were obtained during the study, mean birth weight was 1542 ± 518 g, and mean corrected age was 20.9 ± 4.7 months. Cerebral palsy was found in 11 babies (11.5%). According to Bayley III scores, 13.5% cognitive delay, 19.8% language delay, and 33.3% motor delay rations were detected. A positive correlation was found between GA and motor composite scores (*p* = 0.011). The mean motor composite score was lower in babies with the Apgar score less than 7 at 1st and 5th minutes (*p* = 0.007 and *p* = 0.003) and applied resuscitation in the delivery room (*p* = 0.033). The mean language composite score was found to be higher in babies with antenatal steroid administration (*p* = 0.003). A negative correlation was found between the motor composite score and the oxygen treatment time and mechanical ventilation support time (*p* = 0.001 and *p* = 0.007).

**Conclusion:**

In preterm babies less than 34 weeks, the birth weight, GA, Apgar score, oxygen treatment time, mechanical ventilation support time, and resuscitation in a delivery room were determined to affect the Bayley III motor score. Language development was found better in babies with antenatal steroid administration.

## 1. Introduction

In developing countries, very low birth weight (BW) babies less than 32 weeks of gestational age (GA) have a high risk for neurodevelopmental retardation. This risk is also significantly present in babies 32 to 37 weeks of GA [1, 2]. In recent years, the survival rate of preterm babies has increased with surfactant treatment, antenatal steroid administration, and noninvasive mechanical ventilation supports applied in neonatal care [3]. Today, it is accepted that the criteria of success of newborn intensive care units (NICU) are neurodevelopmental outcomes of babies [4]. Despite the increase in survival rates in developing countries, a similar rate of decrease in the long-term neurodevelopmental retardation rate was not accompanied. The American Academy of Pediatrics published a guide for the monitoring of preterm babies in 2004 [5]. The necessity of neuromotor evaluation of babies with BW below 1500 grams is particularly emphasized. Cerebral plasticity is high in the early period of life. Therefore, it is known that the early detection of neurodevelopmental retardation will increase the positive effect of the interventions [6, 7]. Bayley Scales of Infants and Toddler Development, Third Edition (Bayley-III), which is especially used in the neurodevelopmental of preterm babies has been reviewed. It is composed of cognitive, language, and motor parameters [6]. Bayley III is thought to have a higher capacity to detect developmental delays, especially in preterm infants [7]. In a study from our country, the Bayley II version was reported to have 91% sensitivity and 49% specificity in detecting developmental delay in preterm babies [8]. Although there is no consensus about the cut-off value of the Bayley III test, the limit value is generally accepted to be below 80 to 85 [7, 9].

In this study, with the Bayley III test, we aimed to evaluate the midterm (corrected age of 18 to 24 months) neuromotor development of preterm babies ≤ 34 weeks of GA who were born in our hospital between 2011 and 2014 and treated in our NICU. In addition, it was aimed at determining the risk factors causing a neurodevelopmental delay in babies with a low Bayley III score.

## 2. Methods

All preterm babies ≤ 34 weeks of GA who were born in our hospital between 2011 and 2014 and treated in our NICU were included in the study. Babies born in other centers and referred to our hospital and babies with a congenital major anomaly or congenital metabolic disease were excluded from the study. The study was conducted at Sisli Hamidiye Etfal Children's Hospital in Istanbul which was accepted as reference hospital and NICU that included level II 10 beds and level III 18 beds, with a total of 28 beds. Our hospital has a preterm infant neurodevelopmental follow-up center since 2010.

### 2.1. Study Plan

In the first stage, babies' properties were scanned from the electronic database. Prenatal features (GA and antenatal steroid administration), natal features (gender, BW, height, head circumference, Apgar score at 1st and 5th minutes, whether resuscitation is performed in the delivery room, and mode of delivery), and postnatal features (length of stay in the hospital, oxygen support time, mechanical ventilation support time and continuous positive airway pressure (CPAP) application time, surfactant, caffeine, antibiotic treatment, postnatal steroid treatment, respiratory distress syndrome (RDS), bronchopulmonary dysplasia (BPD), sepsis, necrotizing enterocolitis (NEC), and intraventricular haemorrhages (IVH)) of the patients were recorded.

During the neonatal period, cranial ultrasonography was performed routinely at the following time points: on the first day, on the third day, during follow-up at weeks 1, 2, and 4, and when the newborn was in need. Intraventricular haemorrhages (IVH) were classified into four grades of severity according to Papile. Sepsis was defined as a positive blood culture with positive laboratory finding (elevated C-reactive protein or procalcitonin level) and a need for antibiotic treatment for at least 10 days. Mild/moderate BPD was defined as oxygen treatment (>21%) for at least 28 days and oxygen dependency < 30% at 36 weeks of postmenstrual age. Severe BPD was defined as persisting oxygen requirement (≥30%) and/or positive pressure ventilation or nasal continuous positive airway pressure at 36 weeks of postmenstrual age.

In the second stage, babies at corrected age 18 to 24 months with guardians who agreed to let them participate in the study were called for control. Bayley III was applied to babies by a child development specialist who has a certificate and who does not know the medical history of infants. Bayley III results were recorded on the standard evaluation form [10]. A Bayley composite score of less than 85 was considered as a neurodevelopmental delay. Relationships between composite scores and the characteristics of babies were investigated. All babies underwent neurological examination by pediatricians, and babies with pathological findings were evaluated by a pediatric neurologist. Cerebral palsy was diagnosed according to the European guidelines [11].

## 3. Statistical Analysis

The suitability of the study parameters to normal distribution was evaluated with the Shapiro-Wilks test. Mean, standard deviation, and ratio values were used in descriptive statistics of the data. The one-way ANOVA test was used for the analysis of quantitative independent data. Tukey's HSD test was used to determine the group that caused differences between the groups. The Student *t* test was used for comparing parameters that showed normal distribution between the two groups, and the Mann-Whitney *U* test was used for comparing parameters that did not show normal distribution between the two groups. The chi-square test, Fisher's exact chi-square test, and continuity (Yates) correction were used to compare qualitative data. Pearson correlation analysis was used to examine the relationships between parameters that show normal distribution. *p* values of 0.05 were considered statistically significant. The SPSS 22.0 program was used in the analyses.

## 4. Ethics

This study was approved by the Sisli Children's Hospital human research ethics committee, and written informed consent was obtained from all participants' parents or guardians (number: 2016-158).

## 5. Results

During the study, there were 317 babies born in our hospital with a gestational age of ≤34 weeks. Preterm infants who died (*n* = 71), preterm infants with a major anomaly or metabolic disease (*n* = 36), and preterm infants with incomplete data (*n* = 60) were excluded. The study was completed with 96 babies who met the inclusion criteria after preterm infants whose family cannot be reached (*n* = 30) and preterm infants' family refusing to participate in the study (*n* = 24). Prenatal, natal, and postnatal features of the babies and demographic features during hospitalization in the neonatal period are shown in Table 1. When the babies were included in the study, the mean calendar age was 22.1 ± 3.7 months and the mean corrected age was 20.9 ± 4.7 months. With the neurological examination, 11 babies (11.5%) were diagnosed with cerebral palsy.

The distribution of babies with mean Bayley III composite scores, composite score less than 85, and composite score less than 70 is shown in Table 1. The comparison of the demographic characteristics of the babies with the Bayley III composite score results is shown in Table 2. A significant positive correlation was found between Bayley III motor composite scores and GA and BW. A significantly negative correlation was found between Bayley III motor composite score and mechanical ventilation support time and oxygen requirement time (respectively, *p*: 0.007 and *p*: 0.001) (Table 3).

The comparison of the perinatal characteristics of the babies with the Bayley III composite score results is shown in Table 2. The mean Bayley III motor composite scores were significantly lower in babies 25-26 weeks of GA compared to other GA (*p*_1_ = 0.030, *p*_2_ = 0.048). The mean Bayley III motor composite score was significantly lower in babies with Apgar score of less than 7 at 1st and 5th minutes and babies with applied resuscitation in the delivery room (*p*: 0.007, *p*: 0.003, and *p*: 0.033, respectively). The mean Bayley III language composite score was found to be significantly higher in babies with antenatal steroid administration than babies without antenatal steroid administration (*p* = 0.003).

Laser treatment was applied to 7 babies with ≥stage 3 ROP. It was found that these babies had no effect on cognitive, language, and motor Bayley III composite score results (*p*: 0.830, *p*: 0.896, and *p*: 0.192, respectively). The otoacoustic emission test and clinical Brainstem Evoked Response Audiometry (BERA) test were applied to all infants in the study. Only one baby had a hearing problem in the left ear, but hearing aid support was not required. This situation could not be evaluated statistically. There was no significant relationship between Bayley III composite score results (cognitive, language, and motor) and the diagnosis of the cases (RDS, BPD, sepsis, NEC, and IVH) (*p* > 0.05). There was no significant relationship between the Bayley III composite score results and the use of surfactant, caffeine, and postnatal steroid treatments during hospitalization (*p* > 0.05). There was no relationship between Bayley III composite score results and the mode of delivery, gender, and educational status of parents (*p* > 0.05).

## 6. Discussion

In our study, it was determined that the composite score in 96 preterm babies ≤ 34 GA that we applied Bayley III delayed in cognitive and language development, especially in motor development. The GA, BW, Apgar score of less than 7 at 1st and 5th minutes, applied resuscitation in the delivery room, oxygen requirement time, and mechanical ventilation support time were found to affect motor development. It was found that language development was better in babies with antenatal steroid administration.

Today, the Bayley test is the most used test to evaluate the development of preterm babies [12]. In the literature, the Bayley II test is frequently used [13]. However, in the evaluation of cognitive development with the Bayley II test, cocalculating the cognitive and language areas restricted its effectiveness. Therefore, the Bayley III test was developed in 2006. In Bayley III, the mental development index was divided into cognitive and language areas and the psychomotor development index was divided into gross and fine motor areas [14]. There are few studies in the literature in which the neurodevelopmental evaluation of premature babies was performed with Bayley III. In the studies in which neurodevelopmental evaluations of preterm babies at corrected age 18 to 24 months were performed with the Bayley III test (composite score less than 85), cognitive delay in 6.9-13%, language delay in 21-29.3%, and motor delay in 6.9-16% were reported [15].

In our study, the delay was detected according to Bayley III in 13% of cases in the cognitive development, 19% of cases in the language development, and 32% of cases in the motor development. Motor delay was higher than language and cognitive retardation. The abnormal motor development in most of the cases can be explained by the fact that the babies in our study had a higher GA. Bode et al. [16] evaluated 2-year-old preterm babies with Bayley III, reporting their mean cognitive score as 92 and mean motor score as 97. In another study, 2.5-year-old preterm babies were evaluated, and the mean language scores were 98, the mean cognitive scores were 94, and the mean motor scores were 94 [17]. Lastly, Morgan-Feir et al. [18] reported that motor development delay was 22% by evaluating the motor scale of 1376 babies at corrected age 18 mounts in extremely preterm infants, and Bayley III motor composite score had more clinical benefits than subscale scores. In our study, the mean neurodevelopmental scores of the babies were consistent with the literature in cognitive development and language development, while the motor development was lower than in the literature. Our study supported the knowledge that especially motor delay was more prevalent in preterm infants.

Infections have been shown to negatively affect neurodevelopmental prognosis in very low BW preterm babies. It is known that the risk of developing cerebral movement disorder increases 4 times in babies born from mothers with chorioamnionitis and preterm babies who have had neonatal sepsis [19]. When preterm babies at corrected age 18 to 24 months were evaluated with Bayley III, no significant difference was found between neuromotor delay and clinical sepsis frequency [15]. In contrast, 541 babies less than 28 weeks of GA and proven sepsis with blood culture were evaluated with Bayley II, and the risk of cerebral palsy was reported to be increased in these babies [20]. In our study, there was no significant relationship between Bayley III composite score results and sepsis.

Severe neurodevelopmental sequelae develop in preterm babies with stage 3 and stage 4 IVH, and therefore, these babies are often excluded from neurodevelopmental prognosis studies [21]. Preterm babies at corrected age 20 months with stage 1 or 2 IVH have been shown to have a lower Bayley cognitive development index and higher frequency of major neurological disorders than term babies [22]. In another study, it has been reported that in preterm babies < 37 GA with stage 1 and 2 IVH, it does not have a negative effect on Bayley III scores performed at corrected age 2 years old [1]. In our study, the effect of IVH in preterm babies on Bayley III composite scores was not detected. It is thought that this situation may be caused by two reasons. Firstly, in our study, the number of babies with ≥stage 3 IVH was low, and secondly, our results were not compared with full-term babies.

The antenatal steroid administration in preterm babies reduces the frequency of RDS, mortality, NEC, and IVH [23]. However, some studies have reported that the antenatal steroid administration causes cystic developments in white matter [24]. Erdem et al. [25] reported that 62 babies with BW < 2000 grams and GA < 34 were evaluated with the Bayley test. They found that the rate of antenatal steroid administration was lower in the group with cerebral palsy [25]. In our study, it has been shown that language development was positively affected in preterm babies with antenatal steroid administration.

There is no clear consensus on the effects of postnatal steroid use on neuromotor development. In a study involving many patients, in premature babies who received postnatal systemic corticosteroid therapy in every 1 mg/kg dose of steroid exposure, a reduction of 2.0 points in the mental development index was observed [26]. It was shown that there was no difference in terms of cerebral palsy and behavioural disorders in the 7-year-old evaluation of preterm babies with BPD who received postnatal steroids [27]. In our study, the effect of BPD and postnatal steroid administration on Bayley III composite scores was not detected.

It is demonstrated that Bayley III cognitive, language, and motor scale results are lower in preterm babies than term babies [28]. As the GA and BW decrease, the neurodevelopmental delay rate also increases [1]. In accordance with the literature, in our study, we found that Bayley III composite scores decreased significantly as GA and BW decreased; we also found that motor functions were more affected especially in 25-26 weeks of GA.

In babies with BW less than 1250 grams, caffeine treatment has been shown to reduce the mechanical ventilation support time and the frequency of development of BPD [29]. Schmidt et al. [30] evaluated the 2006 babies between BW 500 and 1250 grams, at corrected age 18 to 21 months and 5 years old. They reported that there was no difference in terms of neurodevelopmental retardation despite the increase in survival rates in babies using caffeine [30]. In our study, there was no positive effect of using caffeine treatment on Bayley III composite scores.

The mechanical ventilation support time has been shown to have a negative effect on Bayley III results in preterm babies [1]. In our study, we found that the mechanical ventilation support time, oxygen requirement time, Apgar score of less than 7 at 1st and 5th minutes, and applied resuscitation in the delivery room had a negative effect on motor scores.

Our study has some strengths. These are the prospective planning of the study, it includes only the babies born in our hospital, and the specialist who performed the Bayley III test performed the tests without knowing the previous diseases of the babies. There are some limitations in our study. These are the study conducted in a single center, the results of the study were not compared with term babies, and the number of patients who came out of follow-up was high.

In developing countries, it is necessary to evaluate the neurodevelopment of preterm babies in the early period and to start the rehabilitation process of risky babies earlier. The frequency of cerebral palsy in preterm babies ≤ 34 weeks of GA at corrected age 18 to 24 months is 11.5%. The GA, BW, mechanical ventilation support time, oxygen requirement time, Apgar score of less than 7 at 1st and 5th minutes, and applied resuscitation in the delivery room affect Bayley III composite scores. Antenatal steroid administration positively affects the language development.

## Figures and Tables

**Table 1:** The prenatal, natal, and postnatal features of the cases and demographic characteristics during hospitalization in the neonatal period.

	Mean ± standard deviation	
Birth weight, g (lower-upper limits)	1542 ± 518	(625-2950)
Gestational age, weeks (lower-upper limits)	30.5 ± 2.5	(24-34)
Antenatal steroid administration, *n* (%)	51	(53.1)
1st minute Apgar score < 7, *n* (%)	54	(56.3)
5th minute Apgar score < 7, *n* (%)	12	(12.5)
Mode of delivery, cesarean, *n* (%)	86	(89.4)
Gender, female, *n* (%)	50	(52.1)
Diseases at hospitalization		
Respiratory distress syndrome, *n* (%)	27	(28.1)
Bronchopulmonary dysplasia, *n* (%)	20	(20.8)
Mild	17	(17.7)
Moderate	3	(3.1)
Sepsis, *n* (%)	20	(20.8)
Necrotizing enterocolitis, *n* (%)	25	(26)
Stage 1	20	(20.8)
Stage 2	5	(5.2)
Intraventricular haemorrhages, *n* (%)	24	(25)
Stage 1	16	(16.6)
Stage 2	4	(4.2)
Stage 3	4	(4.2)
Treatments at hospitalization, *n* (%)		
Oxygen support	88	(91.7)
Mechanical ventilation support	42	(43.8)
CPAP	68	(70.8)
Surfactant	27	(28.1)
Caffeine	45	(46.9)
Antibiotic	88	(91.7)
Postnatal steroid	20	(20.8)
Hospitalization features (lower-upper limits)		
Length of stay in hospital, day	40 ± 28.9	5-166
Oxygen requirement time, day	20.1 ± 27.3	1-166
Mechanical ventilation time, day	10.5 ± 17.4	1-83
CPAP implementation time, day	5.6 ± 6.3	1-33
Duration of antibiotic therapy, day	14.3 ± 11.8	3-74
Bayley III composite scores (lower-upper limits)		
Cognitive scores	93 ± 11.5	55-120
Language scores	93.4 ± 12	59-118
Motor scores	88.5 ± 12.2	46-118
	Composite score < 85 accepted as delay	Composite score < 70 accepted as delay
Abnormal cognitive results, *n* (%)	13 (13.5)	2 (2.1)
Abnormal language results, *n* (%)	19 (19.8)	2 (2.1)
Abnormal motor results, *n* (%)	32 (33.3)	3 (3.1)

CPAP: continuous positive airway pressure.

**Table 2:** The comparison of the demographic characteristics of the babies with the Bayley III composite score.

Characteristic factors	Bayley III composite scores (mean ± standard deviation)		
Gestational age, weeks	Cognitive	Language	Motor
25-26 weeks (*n*: 11)	86.8 ± 10.3	88.9 ± 10.6	78.7 ± 15.5
27-28 weeks (*n*: 5)	99 ± 16.4	89.2 ± 18.6	89.6 ± 11.1
29-30 weeks (*n*: 19)	93.7 ± 14	96.8 ± 15.1	86.5 ± 13.6
31-32 weeks (*n*: 42)	93.2 ± 10.6	92.6 ± 11.3	90.6 ± 10.5
33-34 weeks (*n*: 19)	93.7 ± 9.6	95.3 ± 8.3	91.2 ± 10.5
*p*	0.322	0.359	0.040
Antenatal steroid administration			
Yes	91.4 ± 12.3	89.5 ± 11.6	86.3 ± 12.1
No	94.3 ± 10.7	96.7 ± 11.5	90.5 ± 12.1
*p*	0.220	0.003	0.098
1st minute Apgar score			
<7	92.6 ± 12.9	92.1 ± 13.9	85.7 ± 13.8
≥7	93.5 ± 9.5	94.9 ± 8.9	92.1 ± 8.8
*p*	0.712	0.235	0.007
5th minute Apgar score			
<7	89.6 ± 14.7	88.6 ± 10.8	78.8 ± 14.6
≥7	93.4 ± 11	94 ± 12.1	89.9 ± 11.3
*p*	0.278	0.142	0.003
Resuscitation in delivery room			
No	92.5 ± 10.1	94.1 ± 101	91.2 ± 10.3
Yes	93.4 ± 12.8	92.6 ± 13.7	85.9 ± 13.5
*p*	0.685	0.555	0.033

**Table 3:** Relationship between Bayley III composite score and risk factors.

Case characteristics	Bayley III composite scores					
	Cognitive		Language		Motor	
	*r*	*p*	*r*	*p*	*r*	*p*
Birth weight, g	0.088	0.395	0.059	0.569	0.230	0.024
Gestational age, weeks	0.088	0.396	0.106	0.304	0.260	0.011
Oxygen requirement time, day	-0.188	0.079	-0.203	0.057	-0.338	0.001
Mechanical ventilation time, day	-0.255	0.103	-0.241	0.124	-0.412	0.007

## Data Availability

The database of the study of SPSS file used to support the finding of this study are available from the corresponding author upon request.

## References

[1] Do C. H. T., Kruse A. Y., Wills B. (2020). Neurodevelopment at 2 years corrected age among Vietnamese preterm infants. *Archives of Disease in Childhood*.

[2] Vohr B. (2014). Neurodevelopmental outcomes of extremely preterm infants. *Clinics in Perinatology*.

[3] Horbar J. D., Carpenter J. H., Badger G. J. (2012). Mortality and neonatal morbidity among infants 501 to 1500 grams from 2000 to 2009. *Pediatrics*.

[4] Stephens B. E., Vohr B. R. (2009). Neurodevelopmental outcome of the premature infant. *Pediatric Clinics of North America*.

[5] (2011). Follow-up care of high-risk infants. *Pediatrics*.

[6] Spencer-Smith M. M., Spittle A. J., Lee K. J., Doyle L. W., Anderson P. J. (2015). Bayley-III cognitive and language scales in preterm children. *Pediatrics*.

[7] Vohr B. R., Stephens B. E., Higgins R. D. (2012). Are outcomes of extremely preterm infants improving? Impact of Bayley assessment on outcomes. *The Journal of Pediatrics*.

[8] Saldir M., Sarici S. U., Bakar E. E., Özcan O. (2010). Neurodevelopmental status of preterm newborns at infancy, born at a tertiary care center in Turkey. *American Journal of Perinatology*.

[9] Kaya-Kara Ö., Kerem-Günel M., Yiğit Ş. (2019). Correlation of the Bayley scales of infant-toddler development-3rd edition and neuro-sensory motor assessment in preterm infants during the first year of life. *The Turkish Journal of Pediatrics*.

[10] Bayley N. (2005). *The Bayley Scales of Infant Development*.

[11] Surveillance of Cerebral Palsy in Europe (SCPE) (2000). Surveillance of cerebral palsy in Europe: a collaboration of cerebral palsy surveys and registers. Surveillance of Cerebral Palsy in Europe (SCPE). *Developmental Medicine and Child Neurology*.

[12] Bayley N. (2006). *Bayley Scales of Infant Development III*.

[13] Schlapbach L. J., Ersch J., Adams M., Bernet V., Bucher H. U., Latal B. (2010). Impact of chorioamnionitis and preeclampsia on neurodevelopmental outcome in preterm infants below 32 weeks gestational age. *Acta Paediatrica*.

[14] Moore T., Johnson S., Haider S., Hennessy E., Marlow N. (2012). Relationship between test scores using the second and third editions of the Bayley scales in extremely preterm children. *The Journal of Pediatrics*.

[15] Fernandes L. V., Goulart A. L., Santos A. M., Barros M. C., Guerra C. C., Kopelman B. I. (2012). Neurodevelopmental assessment of very low birth weight preterm infants at corrected age of 18-24 months by Bayley III scales. *Jornal de Pediatria*.

[16] Bode M. M., D'Eugenio D. B., Forsyth N., Coleman J., Gross C. R., Gross S. J. (2009). Outcome of extreme prematurity: a prospective comparison of 2 regional cohorts born 20 years apart. *Pediatrics*.

[17] Chapieski M. L., Evankovich K. D. (1997). Behavioral effects of prematurity. *Seminars in Perinatology*.

[18] Morgan-Feir M., Abbott A., Synnes A., Creighton D., Pillay T., Zwicker J. G. (2019). Comparing standardized and parent-reported motor outcomes of extremely preterm infants. *Children (Basel)*.

[19] Wheater M., Rennie J. M. (2000). Perinatal infection is an important risk factor for cerebral palsy in very-low-birthweight infants. *Developmental Medicine and Child Neurology*.

[20] Schlapbach L. J., Aebischer M., Adams M. (2011). Impact of sepsis on neurodevelopmental outcome in a Swiss National Cohort of extremely premature infants. *Pediatrics*.

[21] Neubauer A. P., Voss W., Kattner E. (2008). Outcome of extremely low birth weight survivors at school age: the influence of perinatal parameters on neurodevelopment. *European Journal of Pediatrics*.

[22] Patra K., Wilson-Costello D., Taylor H. G., Mercuri-Minich N., Hack M. (2006). Grades I-II intraventricular hemorrhage in extremely low birth weight infants: effects on neurodevelopment. *The Journal of Pediatrics*.

[23] Roberts D., Brown J., Medley N., Dalziel S. R. (2017). Antenatal corticosteroids for accelerating fetal lung maturation for women at risk of preterm birth. *Cochrane Database of Systematic Reviews*.

[24] Ghotra S., Vincer M., Allen V. M., Khan N. (2019). A population-based study of cystic white matter injury on ultrasound in very preterm infants born over two decades in Nova Scotia, Canada. *Journal of Perinatology*.

[25] Erdem G., Bakar E. E., Yiğit Ş., Turanli G. (2006). Hacettepe Üniversitesi Hastanesi Yenidoğan Yoğun Bakım Ünitesi’nde izlenen prematüre bebeklerin nörogelişimsel izlemi. *Çocuk Sağlığı ve Hastalıkları Dergisi*.

[26] Wilson-Costello D., Walsh M. C., Langer J. C. (2009). Impact of postnatal corticosteroid use on neurodevelopment at 18 to 22 months' adjusted age: effects of dose, timing, and risk of bronchopulmonary dysplasia in extremely low birth weight infants. *Pediatrics*.

[27] Wilson T. T., Waters L., Patterson C. C. (2006). Neurodevelopmental and respiratory follow-up results at 7 years for children from the United Kingdom and Ireland enrolled in a randomized trial of early and late postnatal corticosteroid treatment, systemic and inhaled (the open study of early corticosteroid treatment). *Pediatrics*.

[28] Serenius F., Källén K., Blennow M. (2013). Neurodevelopmental outcome in extremely preterm infants at 2.5 years after active perinatal care in Sweden. *JAMA*.

[29] Davis P. G., Schmidt B., Roberts R. S. (2010). Caffeine for apnea of prematurity trial: benefits may vary in subgroups. *The Journal of Pediatrics*.

[30] Schmidt B., Anderson P. J., Doyle L. W. (2012). Survival without disability to age 5 years after neonatal caffeine therapy for apnea of prematurity. *JAMA*.

